# “I Always Vet Things”: Navigating Privacy and the Presentation of Self on Health Discussion Boards Among Individuals with Long-Term Conditions

**DOI:** 10.2196/jmir.6019

**Published:** 2016-10-13

**Authors:** Ellen Brady, Julia Segar, Caroline Sanders

**Affiliations:** ^1^ Centre for Primary Care University of Manchester Manchester United Kingdom; ^2^ NIHR School for Primary Care Research, Manchester Academic Health Sciences Centre University of Manchester Manchester United Kingdom

**Keywords:** privacy, ethics, research ethics, informed consent, patients, social support

## Abstract

**Background:**

The ethics of research into online communities is a long-debated issue, with many researchers arguing that open-access discussion groups are publically accessible data and do not require informed consent from participants for their use for research purposes. However, it has been suggested that there is a discrepancy between the perceived and actual privacy of user-generated online content by community members.

**Objective:**

There has been very little research regarding how privacy is experienced and enacted online. The objective of this study is to address this gap by qualitatively exploring the expectations of privacy on Internet forums among individuals with long-term conditions.

**Methods:**

Semistructured interviews were conducted with 20 participants with myalgic encephalomyelitis/chronic fatigue syndrome (ME/CFS) and 21 participants with type 1 and 2 diabetes mellitus, and were analyzed using thematic analysis. Participants were recruited via online and offline routes, namely forums, email lists, newsletters, and face-to-face support groups.

**Results:**

The findings indicate that privacy online is a nebulous concept. Rather than individuals drawing a clear-cut distinction between what they would and would not be comfortable sharing online, it was evident that these situations were contextually dependent and related to a number of unique and individual factors.

**Conclusions:**

Interviewees were seen to carefully manage how they presented themselves on forums, filtering and selecting the information that they shared about themselves in order to develop and maintain a particular online persona, while maintaining and preserving an acceptable level of privacy.

## Introduction

Rapid advances in technology and Internet use have led to an increasingly evolving body of research and practice in the area of eHealth. In particular, there has been a wealth of patient-centered systems and services, such as the growth of informal support systems via online patient communities [1,2]. In line with this growth, there has been an equivalent focus on these online communities by researchers. However, despite the growing use of user-generated content as data by researchers, less attention has been paid to the ethical considerations surrounding this research. This paper aims to contribute to discussions in this area by exploring the notions of “public” and “private” spaces among individuals with long-term conditions.

The ethics surrounding the study of Web-based interactions is a long-debated issue. For example, King [3] argues that it is unnecessary to receive permission from a virtual community to conduct research based on messages generated in publicly available spaces, as long as certain criteria surrounding privacy are adhered to, for example, removing all references to the name and type of the groups. Similarly, Reid [4] mentions that once participants in a multi-user dungeon (MUD), a type of Internet forum, learned the nature of her research, they began to “manufacture quotable quotes” (p 171), leading her to conclude that nondisclosure of her research was not only justified but also essential.

In addition, many researchers make distinctions between degrees of public and private spaces online. Many of the forums used in health care research do not require any subscription or registration in order to access the messages; thus, it has been suggested that members of such forums are not likely to view the discussion boards as a “private place” online [5]. For example, Elwell [6], in studying forums used by adolescents with cancer, justified the lack of informed consent from forum participants on this basis, saying:

Ethical issues associated with the present project include the issue of informed consent, as the adolescents who posted messages to the computer-mediated support group are not aware that their messages are being used for research purposes, so thus have not formally consented. However, in the present study an online support group was chosen that did not require subscription or registration in order to access the messages, thus it is argued that messages posted to the computer-mediated social support group are indeed accessible to the public and thus informed consent from the adolescents in this instance is not required.

p 239

Although this argument is frequently made in the study of online forums [7-9], ethical concerns continue to be extensively discussed, reflecting a common discomfort with observational research online [10]. Some early attempts were made to establish a series of ethical guidelines around the Internet as a source of data, notably the 2002 recommendations from the Association of Internet Researchers (AoIR). These guidelines were updated in 2012 to acknowledge the evolving field of Internet ethics. As the guidelines themselves acknowledge, rather than representing a strict code of behavior, they merely serve to “emphasize processes for decision making and questions that can be applied to ever-changing technological contexts” [11]. The recommendations cover a number of topics that are beyond the scope of this paper, but of key relevance to the present research is that they highlight the nebulous notion of privacy. In particular, they outline how social, academic, and regulatory distinctions between public and private are not likely to be applicable in the context of the Internet and social media.

### Public and Private Spaces Online

It is first necessary to consider what current research exists on these topics. The AoIR recommendations highlight the potential for discrepancies to exist between the actual privacy and the perceived privacy of online content. For example, despite forum content being publically accessible and available to anyone with a Web connection, it is possible that the creators of the content may perceive that the information, experiences, and opinions that they share online are being disseminated in a private space. This may have particular resonance for health-related forums, where the topics under discussion may have a particular emotive significance [12]. As Daker-White et al [12] highlight, the knowledge that their words and experience could potentially be shared and disseminated could have an impact on participants’ posting style or even discourage them from posting.

The potential contradictions between notions of public and private are covered at length by boyd and Marwick [13]. In this article, they describe a scenario where images from teenagers’ Facebook pages were used in an educational lecture on Internet safety by educators and law enforcement officials in the United States. Despite students being aware that the information and pictures that they shared on Facebook were public or relatively public, their expectations of privacy included an expectation that their profiles would not be accessed and shared without their prior knowledge and consent. Students reacted angrily, describing the lecture as “a violation of privacy” (p 6).

The authors argue that rather than representing a contradictory stance, this perception is in line with typical social norms around public engagement [13]. They suggest that expectations of privacy online mirror expectations of privacy offline—one would not expect a conversation held in a public restaurant to be overheard and broadcast, despite the knowledge that the conversation *can* be overheard [14]. Indeed, early research into computer-mediated communication indicated that individuals often self-disclose very personal information online that they would not be willing to reveal offline, known as the “online disinhibition effect” [15].

Supporting this notion, other researchers have pointed to apparent discrepancies in Internet users’ perceptions and expectations of privacy. Bassett and O’Riordan [16] highlight an example in which lesbian, gay, bisexual, and transgender forum users’ constructions of privacy online, and their expected levels of confidentiality, safety, and freedom, were sharply divergent from both the actual levels of privacy and access and the description of the site and forum provided by the website owners. This indicates that despite signals to the contrary, individuals involved in online discussion groups may view the spaces that they occupy online as safe spaces, unlikely to be accessed or disseminated by outsiders.

As Hogan [17] indicates, expectations of privacy online do not necessarily indicate that individuals are sharing information that they wish to remain hidden. Rather it suggests that, when information is shared, the people with whom the information has been explicitly shared (ie, forum users, Facebook friends, members of an email list) are considered to be contextually appropriate for the specific information [18]. This notion of contextual integrity [18,19] holds that conceptions of privacy are shaped by the norms of the contexts in which the information is shared. Instead of utilizing a strict public/private dichotomy, Nissenbaum [18,19] proposes that individuals exist in a plurality of realms, each with different guidelines outlining how to act and interact. Privacy is considered to be violated when norms specific to a particular context are violated, such as norms about what information is appropriate to divulge in a given situation or how it is appropriate for that information to be distributed [18].

For example, within a health care consultation, it is considered appropriate for an individual to share information about his or her physical condition with a doctor. However, if the doctor were to reciprocate by divulging information about his or her own physical state, that would not be seen as appropriate. Similarly, although an individual may expect a doctor to share information about his or her condition with a practice nurse, if required, it is likely that distributing that same information to the doctor’s family and friends would be receive an extremely negative reaction from the patient [19]. Although the information being shared in all cases may be virtually identical, the alterations in context and audience result in privacy norms being violated.

However, as Marwick and boyd [20] point out, this model presumes that the individual at the center of the scenario is fully aware of the social context surrounding their disclosure. In order to navigate privacy online, individuals must have the technological expertise to operate their medium of sharing information, as well as the knowledge and skills to influence how information flows in an online context and how it is interpreted within that context. Instead, they propose a model of networked privacy, which draws on social media research to argue that information norms are co-constructed by participants and are constantly shifting due to variations in social norms and technological skills among individuals. This further highlights the complexities of privacy online, and suggests that a blanket approach toward particular media as “public spaces” or “private spaces” may be problematic.

From a research perspective, Hudson and Bruckman [21] reported that many of the chat rooms they entered as part of their study responded negatively to the presence of researchers. In the majority of cases, the researchers were “kicked out” or banned from participating in the space. Comments from some groups indicated that they viewed the publically accessible chat rooms as private spaces and were unwilling to tolerate the use of the content for research purposes. Although this is in line with findings from boyd and Marwick [13] and Bassett and O’Riordan [16], it does have potential implications for the use of user-generated content and particularly forum content as data. Specifically, it contradicts the assumption that publically accessible spaces online are seen as public spaces by participants [5,6] and, therefore, do not require informed consent from users.

There has been a growing use of forums in research, particularly health research and, to a lesser extent, debate and dialog around the ethical implications of this practice [22-24], but there has been a dearth of research directly exploring forum participants’ perceptions and expectations of public and private spaces online. Bond et al [10] interviewed users of online diabetes discussion boards. Although the participants were generally supportive of the use of forum data for research purposes, citing the need for the voices of individuals with diabetes to be heard, there was less of a consensus about the specifics of using the data.

Despite many participants acknowledging that their posts were publically available and, therefore, ultimately in the public domain, a number were uncomfortable with their words being used without their consent. In particular, the use of direct quotes was controversial, with interviewees expressing concern that they may be identifiable from the quotes [10]. Although these findings provide some insight into the views and perceptions of forum users, the brief nature of the research offers little clarity around the topic and indicates the need for further research.

Given this, it seems particularly important to give consideration to concepts of public and private spaces within Internet forums, specifically within health-related discussion forums. As the AoIR guidelines suggest, this will by no means result in a strictly defined delineation between the two concepts. Factors such as the level of access available, the number of forum users, and individual forum guidelines and norms will all likely play a role in establishing the boundaries between public and private spaces [5]. Nevertheless, an exploration of these concepts within specific conditions may provide a useful case study of the notions of “public” and “private” in practice.

In this paper, perceptions of privacy on Internet forums are explored by drawing on analysis of qualitative data gathered as part of a broader study into the use of online discussion boards by individuals with long-term conditions. To do this, semistructured qualitative interviews were conducted with UK-based individuals recruited from two population groups. One sample consisted of individuals with type 1 and 2 diabetes mellitus. Diabetes affects more than 5% of the British population and has been highlighted by the National Health Service as a key focus of the effort to improve chronic disease management in the United Kingdom [25]. The other sample consisted of individuals with myalgic encephalomyelitis/chronic fatigue syndrome (ME/CFS), which is characterized by fatigue, pain, and impaired cognitive functioning, and affects up to 100,000 people per year in the United Kingdom [26].

## Methods

A qualitative approach was selected as it allowed for an exploration of the opinions and perspectives of individuals with long-term conditions. A broad interview schedule was developed, which explored the role and nature of online support for those with long-term conditions. Interviewees were given space to express their own opinions and ideas; in many cases, their responses shaped the order and structure of the interview [27]. In addition to the broader health-related questions, interviewees were asked about their views of online support groups as public or private spaces and their concepts of audience when sharing and receiving information and experiences online. This was also explored via a vignette (seen in Textbox 1), which was adapted from existing forum data. It was intended that the use of a vignette would enable participants to consider themselves in the place of the character [28].

**Vignette used during the interview process.**

Rachel has ME/CFS and regularly posts in a forum used by other people with similar symptoms. She is concerned that the forum can be viewed by anyone, not just the people who contribute to it. She starts a thread on the forum to discuss this and to see if other people feel the same way.

**Rachel:**

*"Some people are revealing some quite intimate info, and I myself often forget that the forum is open to outsiders to view."*

Here are some of the responses that Rachel receives.

**Sarah:**

*"Not too bothered by this as I have never said anything that I would not be prepared to share with the whole world. I tend to think that the more people that know of the devastation that is caused by this illness, the better. I would like to think that some of the stuff is read by the medical profession—though I think not!!!"*

**Jane:**

*"Hi, I’ve just seen this thread and am rather concerned and wondering whether not to post anymore, to be honest. That’s not just this forum but a lot of open forums too.*

*People need to realize that as we all get so very down, we may say things on here that we wouldn’t say to family and friends and maybe it’s rather personal.*

*"I shall be having a think as to whether I shall post on here for a while now."*

What do you think about what people have said to Rachel?

What would you say to Rachel?

Do you have anything else to add?

### Participants

A total of 41 participants completed interviews, 20 with ME/CFS, 12 with type 1 diabetes, and nine with type 2 diabetes. Interviewees were drawn from across the United Kingdom and the majority of the respondents were female (n=28), with a mean age of 50 (range 18-82) years. To ensure that a range of perspectives were considered, recruitment took place both online and offline. Interviewees were recruited through online and offline sites, such as Internet forums, face-to-face support groups, email lists, and research networks.

Participants were offered the option of face-to-face or phone interviews with the researcher; many (n=29) chose to participate by phone. All interviewees described themselves as white. Notably, the majority of participants (71%, 29/41) had completed at least a higher education degree or equivalent. The latest census data suggested that in 2011, just 27% of the population of England and Wales had received a degree or higher [29], indicating that participants in this study were educated to a higher level than the general British population.

### Data Analysis

Interviews were recorded and transcribed verbatim, including participants’ responses to the vignette. The anonymized interview transcripts were imported into a qualitative data analysis computer software package (ATLAS.ti version 7) in order to carry out the analysis. It should be noted that the use of a software package merely provided a tool to organize and review the data during the analysis process, rather than offering an objective method of analysis [30]. Each transcript was read through several times and notes were made in order to make note of preliminary connections between interviewees.

A thematic method of analysis was employed, with a view to examining comparisons and contrasts across participants and within cases. Thematic analysis was chosen because it provided a flexible approach to analyzing qualitative data and involves identifying themes in a body of data [31]. Themes were considered to capture something important about the data and to represent a level of patterned response or meaning within a dataset. This process allows the development of a conceptual scheme, which enables further interrogation of the data [32].

The analysis followed an iterative process. A coding frame was devised comprising the initial themes identified within the data. Following this, the data were coded according to these themes. Initially, these codes were broadly descriptive, and related directly to the content of interviewees’ transcripts, rather than subtle nuances within the data. For example, references to an interviewee’s family were coded as “family” and so on. As coding continued, categories were further refined into subcategories or aggregated to form higher-level categories because the initial coding frame did not sufficiently capture the complexities of the data. The coding frame was continually revised and transcripts were reviewed on an ongoing basis to ensure that additional codes were applied.

### Ethics

Ethical approval was granted by the University of Manchester research ethics committee. Any identifying information was removed from the interview transcripts and all participants were assigned pseudonyms. Each participant was provided with an information sheet and encouraged to contact the researcher with any questions both before and after the interview. Signed consent was received from all participants; for telephone interviews, the consent form was mailed in advance along with a stamped addressed envelope to return the signed form to the researcher.

## Results

Participants experienced privacy online as a complex and nuanced process. Interviewees were seen to carefully manage how they presented themselves on forums, selecting the information that they shared about themselves and where this information was shared in order to develop and maintain a particular online persona, while preserving their privacy. The context in which information was shared influenced users’ decisions about what to disclose and not to disclose online, with individuals adapting what they shared online in order to ensure it was appropriate for the broadest possible audience. In addition, the value of sharing personal information online for fellow patients and health care professionals was consistently highlighted by participants. The process of navigating privacy online is outlined in the following sections.

### Presentation of Self Online

For many participants, maintaining their anonymity online was a pivotal aspect of their usage of forums. As “Gemma” (type 2 diabetes, 31-35 years) described it, the diabetes forum she used was “my place and that’s my space to talk about things.” Although both her parents had been diagnosed with diabetes, she had not told them about her own diagnosis. In addition, she had been extremely reluctant to share that information with family and friends: “The only people that know that I’m diabetic is my husband and my best friend, I haven’t told my family and friends, even though my family are, even though my parents are diabetic, I haven’t told them.” As a result, her diabetes was an intensely personal experience, shared with the forum and a select few acquaintances in real life. This meant that anonymity was a pivotal aspect of her condition (“It would really, really bother me if people found that information, if by googling my name, it came up with all this information, I would be devastated really”) and she was unwilling to disclose information about her diabetes to her real-life acquaintances (“I would stop using it”).

This suggests that, for some individuals, online discussion groups provide them with a “safe space” in which they could access support away from their real-life support networks [33,34]. For these interviewees, however, the safety of the space was tempered by the awareness that their words may be disseminated among a wider audience than they intended. For others, having an identifiable online presence was something that they had consciously chosen, rather than attempting to remain anonymous online. This was the case for “Louise” (type 1 diabetes, 31-35 years), who regularly blogged about her experiences with type 1 diabetes. She described how she had decided to blog as herself rather than an anonymous individual because she had already been active in the diabetes community for a number of years and, as a result, had a “good network” of peers. By naming herself on her blog, she felt that she was able to “talk freely” about herself, and describe her own situation and experiences, rather than hiding behind an “anonymous persona.” However, she acknowledged the impact that this lack of anonymity had had on her online communications. She described how having her words linked to her offline identity made her consider how they were likely to be viewed by an audience, such as a potential employer: “I always vet things with the idea of, OK, would I be happy to discuss this in an interview.”

Despite Louise’s willingness to identify herself online, her reference to her employer indicates that she was managing her performance and persona online. As research on social media suggests [17,35,36], individuals will adapt the information that they share online in order to cater to the “lowest common denominator” (ie, cater to the broadest audience possible). As a result, Louise, Gemma, and many other interviewees took care to ensure that only certain aspects of themselves were represented online [36]. This self-censorship extended beyond forum participants to members of their social networks. Interviewees spoke about how, while they were happy to share their own experiences online, they avoided sharing personal information about their children or other family members. Participants were conscious that although they could control the level of information that they provided about themselves online, others may not be happy to have information shared about them:

When I mentioned about my son going through a difficult time...I don’t mean, I don’t mention him, what I mean is, I don’t mention the difficulties he went through and what it was to do with or anything.

Joan, ME/CFS, 56-60 years

This sentiment was echoed by Louise. Although she frequently blogged about her experiences with diabetes, she made a decision not to disclose her experiences with fertility treatments. She was conscious that sharing information and experiences regarding in vitro fertilization (IVF) and diabetes could be of value to others, she felt that the information was “too personal, too vulnerable” to share, despite the potential benefits. Interestingly, she later wrote about her experiences with IVF after she became pregnant, indicating that her desire for privacy was shaped by the need to control the context in which the information was shared rather than the information itself [18-20,35]:

That was quite a tough decision because in a way I wanted to share what we were going through because no one writes anything about IVF and diabetes, that’s such a niche problem. It’s very hard to find good information about it. But I just didn’t feel I could expose that kind of thing to the Internet. That was too personal, too vulnerable, especially when we were in the middle of it. Now, I have written some stuff about it looking back, so it’s interesting. I don’t censor much of what I put online, but there are bits that I do.

Louise, type 1 diabetes, 31-35 years

### The Value of Sharing Information Online

Despite participants’ perceptions of forums as public spaces, or perhaps because of these perceptions, many interviewees reported that they saw a value in sharing their experiences within a public arena. Although participants acknowledged that their words could be accessed by those outside of the immediate audience, this was seen as a pivotal aspect of sharing experiences online. This was particularly prevalent among those with ME/CFS, many of whom felt that the Internet and Internet forums enabled individuals to describe the daily realities of living with ME/CFS. “Michelle” (ME/CFS, 41-45 years) reported that she shared experiences online in order to address those who may have family or friends with ME/CFS. By sharing her own experiences online, she attempted to legitimize the experiences of others by validating their feelings and symptoms:

If you have the partners, or the family watching this kind of website to understand better, if they can see that something their daughters, or whoever, told them about and they can see it said by someone else, maybe they will understand better.

Michelle, ME/CFS, 41-45 years

For other participants, the notion that sharing information and experiences online could be of value to health care professionals was highlighted. In response to the vignette, “Nicole” (ME/CFS, 26-30 years) suggested that medical professionals accessing Internet forums could increase their understanding around ME/CFS, which could translate into improved health care for patients: “Sometimes I would like some people from the medical profession to read it and to understand, because the understanding around chronic fatigue is terrible.” This was of similar importance to “Mark” (type 1 diabetes, 41-45 years), who felt that medical professionals accessing Internet forums for individuals with diabetes would not only lead to increased understanding around diabetes, but would also illustrate to professionals the potential benefits to individuals accessing online support: “I think there needs to be a bit of a sea change in some minds of health care professionals, that it’s not actually all bad but that it is a positive experience and it can really help.” Indeed, this echoes recent trends among clinicians, with suggestions that the “cloud of patient experience” online may provide valuable insights into care unfiltered by health care professionals, researchers, or academics [37,38].

### Curating the Information Shared Online

In addition to individuals filtering what information they shared online in order to manage their online persona [36,39], interviewees also described how they drew distinctions between where to share their experiences, advice, and information with peers and where not to share this information. The “permanent” nature of Internet forums, some of which did not allow users to delete their posts after a certain period of time, was discussed by many participants, with some reporting that this made them less likely to discuss particular topics in this arena. This led to forum members utilizing other methods of communication, such as live chat, instant messaging, emails, or private messages. Rather than making a blanket distinction about what personal information or data to share and not share online, participants instead considered the context in which information was shared and who was likely to access this information [18-20,35].

For participants, this often meant seeking out spaces online, which were not fully open or publically accessible in order to share information that they considered to be very personal. For example, “Lesley” (type 2 diabetes, 56-60 years) described how she used the live chat on the diabetes forum that she was a member of, which enabled her to exchange instant messages with other forum members. Crucially, using live chat meant that the conversation was not stored afterward and was not publically accessible, even by those who were registered forum members: “If you go on live chat, it’s there, and then when you go off, it’s gone, if you know what I mean, it’s not stored anywhere for anyone else to come and read.” She used this option to talk regularly to other forum members who she considered to be friends, sharing information about their day-to-day experiences with diabetes: “You know, ‘oh, my blood sugar’s up today,’ ‘oh, I’ve had such a thing for my tea and I shouldn’t have done,’ and you know, things like that, what we’ve eaten, the nitty-gritty bits, that’s what we tend to do.” And they also shared more personal information that may not be appropriate for discussion on the forum: “And then we talk personally, you know, how’s it going at home, are you OK, you know, have you been to work today, things like that that you wouldn’t put on the forum because that’s very personal.”

Despite valuing the privacy that this medium afforded her, Lesley still used the forum to discuss “major problems” about her diabetes over the live chat. She recognized the impact that sharing experiences openly had on other people, and wanted to be able to offer that support to others: “The point of the forum, I think, is to help other people who might be like I was doing and just reading, and don’t want to join, and they want to gain something from your experience.” This emphasizes the value of sharing information online, as discussed previously.

It is important to acknowledge the educational backgrounds of the participants in this study and to consider how this may have influenced individuals’ perceptions of privacy online. Many drew explicitly and implicitly on their level of education or work experience in describing how they navigated Internet forums. Indeed, Papacharissi and Gibson [35] describe privacy online as a form of “luxury commodity” (p 85), arguing that the level of computer literacy required to acquire it is inaccessible to many. For example, “Karen” (ME/CFS, 41-45 years) had a degree in information technology and she felt that this background gave her an advantage when it came to deciding what information to share and what not to share online. Like many other interviewees, she viewed the Internet and Internet forums as public spaces, and this influenced how she interacted with others online: “It’s permanent, it don’t matter what you do with it, it’s up there. So I wouldn’t put anything up there that I wouldn’t want a stranger [to read], do you know what I mean?” She drew a distinction between her experiences and those of her husband, who did not have the same educational background and, as a result, struggled to utilize the Internet in the same way:

I’m lucky in the sense that I’ve actually studied the Internet and I’ve studied computing, so I have a bit more information than maybe say, like, my husband doesn’t have that much information or nous about the Internet, so he’d be likely to worry about things like that more than me and he’d ask me and I’d say, well, you’re alright to do that but not that.

Karen, ME/CFS, 41-45 years

### Online Audiences

Given that the majority of interviewees viewed forums as public rather than private spaces, it is necessary to examine whom they felt they were sharing information with online. Nissenbaum [18,19] suggests that the context in which information is shared influences users’ expectations around privacy. In particular, the people with whom information has been shared have been considered appropriate recipients for the specific information. For some of the interviewees recruited via offline sources, the public nature of forums was a barrier to them utilizing forums to share personal information online. In line with the expectation that forums were public spaces, for many individuals, their concept of audience extended outside the members who were actively participating in the forum. For example, interviewees spoke about sharing information online in light of the possibility that their words could be accessed by family and friends.

Illustrating the concept of the lowest common denominator [17], Michelle described how she considered the perspectives of her husband and parents in her interactions online. Although she did not think it was likely that they would access an Internet forum, the awareness that they had the ability to read what she wrote meant that she ensured that she could “justify” what she said to them:

I don’t think my husband is reading it, but maybe he is...I think, yeah, he wasn’t supportive, or anything, I would put it in writing if it were true and if he knows about it, because I’ve talked about it with him.

Michelle, ME/CFS, 41-45 years

For other participants, their concept of audience extended outside their family and friends to include outside parties. This was illustrated by “Susan” (ME/CFS, no age given), who blogged about her experiences with ME/CFS. She was particularly concerned about protecting her identity online because she worried that her online activity would be seen as evidence that she was fit for work by the Department of Work and Pensions (DWP) and would have an impact on the benefits and allowances to which she was currently entitled:

I’ve not actually put my name on the blog...that’s because really of potential criticism from somebody like the DWP, because, you know, if they see I’ve written that blog and I’ve got that amount of information on it. They’ll turn around and say, well, crikey, you’re fit to work.

Susan, ME/CFS, no age given

However, it should be noted that this awareness of external audiences was not present throughout the entire sample. Some interviewees held a different perception of forums, viewing them as a more private and personal space. Like Karen, many participants expressed concerns that although they were aware of the public nature of Internet forums, others may not be as savvy as them and, as a result, may experience difficulties navigating concerns around privacy and anonymity online. For example, “Michael” (ME/CFS, 66-70 years) described how he had encountered a number of people who had shared information online that he felt was inappropriate:

I’m all for frankness and openness but some of the things that I had read I was surprised that people would have put that information in that domain when you think of who could actually see that and that just concerned me a bit.

Michael, ME/CFS, 66-70 years

Illustrating this, “Jennifer” (ME/CFS, 36-40 years) drew a comparison between sites online where “anyone could read it and anyone could respond” (eg, comments on the BBC website) and ME/CFS forums. By contrast, forums were seen as less of an unknown quantity, with the expectation that there was a mutual understanding and respect among members: “If it was an ME forum, then, yeah, I think it’s nice to know that you can walk into to a space that you’ve chosen to and that you know what you’re walking into.” Despite the fact that both spaces were open-access, online public arenas, Jennifer perceived that discussion boards had a deeper level of privacy.

This suggests support for Nissenbaum’s notion of contextual integrity [18,19]. Although the majority of interviewees viewed open forums as public spaces, there were exceptions to this. Rather than a strict delineation between public and private spaces online, the context in which the information was shared—in this case, a health-related Internet forum—influenced users’ expectations of who could access their words. Participants raised concerns about the supportive nature of online discussion groups and cognitive impairments associated with ME/CFS that could encourage forum users to share information that may be inappropriate or potentially identifying:

People in desperation reach out and other people who’ve been in this cozy environment, this kind of warm room full of friends sharing things openly, forgetting that complete strangers can then just look and read.

Mark, type 1 diabetes, 41-45 years

People with ME, because of the tiredness, etc—I do things now that I wouldn’t dream of doing, just by mistake, I wouldn’t dream of doing when I was well.

Nicole, ME/CFS, 26-30 years

As a result, it is possible that the online context in which individuals perceive they are interacting may not accurately reflect the reality of the situation.

## Discussion

The findings support the notion that privacy online is a nebulous concept. For participants, online discussion boards enabled them to reveal information that was intensely personal and private and that they did not feel comfortable sharing in an offline setting, such as with their family and friends. This suggests that, for some individuals, the forums provided them with a safe space in which they could access support away from their real-life support networks [33,34]. However, this does not mean that the information shared on forums represents an unfiltered expression of forum members’ thoughts and feelings. In keeping with Goffman’s [40] dramaturgical work on identity, participants described a degree of impression management, where they filtered and adapted the information that they shared online in order to create a particular identity for themselves. For many individuals, their adopted online persona was an anonymous one and they spent time censoring and editing what they shared to ensure that their online and offline identities remained separate.

In this way, the findings of this study support previous social media research on the notion of the lowest common denominator, in which individuals adapted what they shared online to ensure it was appropriate for the broadest possible audience [17,35,36]. Interviewees described scrutinizing and modifying their online communications in light of the audiences that they felt could access their words, such as employers, family members, journalists, or government agencies.

In order to remain anonymous online, this self-censorship involved avoiding revealing identifying information such as an individual’s place of home or work. For the majority of interviewees, remaining anonymous online was desirable, supporting previous research that indicated that being able to contact peers anonymously is an important aspect of individuals accessing and receiving support online, particularly for health-related queries [33,41]. Even for individuals who did not maintain an anonymous persona online, there was still a sense of managing and monitoring the words and information that they shared. This suggests that maintaining an identifiable online persona is not merely a direct replication of one’s offline identity. Rather, only some aspects of oneself are presented online. However, as highlighted by Bullingham and Vasconcelos [36], this can be a two-way process. Although some individuals may carefully share aspects of themselves online in order to present a delicately constructed persona, others may in fact offer their “true selves” online, in cases in which their offline self is influenced by societal or family pressure. Within this study, forum users often utilized both aspects of this presentation simultaneously, describing how they took care to present an anonymous online persona, while at the same time sharing their true feelings and experiences with their condition that they would not feel safe or comfortable sharing with their family and friends. In this way, forums provided a space for posters to perform aspects of their identity unconstrained by offline relationships [34,36].

Despite this, there were concerns from participants that certain forum members were not engaged in a sufficient level of identity management online, leading to ineffective attempts at safeguarding privacy. Although all interviewees felt that they themselves were in control of the information that they disclosed and were capable of navigating and negating any privacy concerns online, some expressed doubts that other Internet users were as competent at these tasks. Returning to the notion of the lowest common denominator, participants suggested that for some forum users, their version of the lowest common denominator was an unrealistic one that did not account for the public nature of Internet forums [17]. Suggested reasons for this included the supportive nature of health discussion groups, cognitive impairments or “brain fog” associated with ME/CFS, as well as a lack of experience or education around the Internet and the nature of social media.

Although the digital divide has been frequently discussed in relation to health literacy [42,43], this paper also points to its relevance to online privacy. This has been highlighted within the literature; Papacharissi and Gibson [35] describe privacy online as a luxury commodity, arguing that the level of computer literacy required in order to acquire it is inaccessible to many. Similarly, Osatuyi [44] highlights the link between confidence in Internet skills and privacy, where users who are less confident in their abilities to navigate social media are less likely to engage with these technologies due to concerns about information privacy. As a result, it is important to note that discussions around the use of online health discussion groups by individuals with long-term conditions may relate to those who have successfully navigated these complexities, rather than a wider population.

In addition, the findings illustrate the notion of privacy online as a nebulous concept. Rather than individuals drawing a clear-cut distinction between what they would and would not be comfortable sharing online, it was evident that these situations were contextually dependent and related to a number of unique and individual factors [18,19]. For example, forum users described how they shared certain information using private messaging or online chat facilities rather than posting on a public forum, indicating that their desire for privacy was shaped by the need to control the context in which the information was shared rather than a need to keep the information itself private [20,35]. This suggests that navigating the different spaces and performative “stages” of Internet forums [17,40] requires an awareness of both the social and technical aspects of these forms of social networks [20]. In addition, as Papacharissi and Gibson [35] highlight, there is an inherent difficulty in negotiating privacy in networked social environments that were designed for sharing rather than privacy. Although their argument relates to social media rather than Internet forums, it is evident that parallels can be drawn between the two spaces.

The results indicate that concerns around privacy are perceived as an additional barrier to those with insufficient levels of digital literacy accessing support online. Nutbeam [45] argues that in order for health literacy to occur, individuals are required to have both the confidence and the skills to gather information, understand it, and actively appraise it. Interviewees suggested that the utilization of forums was a complex process and achieving privacy was a difficult yet pivotal aspect of this utilization. Achieving privacy requires an understanding of networked privacy [20] and the role of contextual factors, such as forum norms and the function of the moderators, as well as the technical aspects of navigating around an Internet forum. As a result, maintaining an online persona, which for many of the participants in this study meant remaining anonymous and carefully considering where to share personal information, is at risk of becoming the preserve of a select few [35]. This means that research into the use of health-related forums must consider the impact of inequalities on forum usage and particularly highly contextual and nuanced factors such as privacy. In order to contribute to the body of knowledge in this area, this research highlights the need to examine how privacy is situated within online literacy. In addition, this has implications for those involved in the creation, curation, or moderation of online spaces because it emphasizes the need to cater for a broad range of users within health-related forums.

Finally, this research aimed to provide some guidance on the ethics of conducting research into online spaces. It was concluded that forums are predominately viewed as public spaces, and forum members adapt what they share online in light of this perception. This is similar to research on Facebook, which indicates that although there are privacy concerns about the medium, information posted on Facebook is tailored toward a broad social audience [46]. This has implications for the use of forum posts as data because it suggests that in the case of health discussion boards, participants generally expected that what they shared online would be accessed by a broader audience beyond those whom they were directly interacting with. However, the findings of this study are likely to be highly context specific and this should not be taken as a blanket suggestion that will apply to all health discussion boards.

## Abbreviations

AoIR: Association of Internet Researchers
DWP: Department of Work and Pensions
ME/CFS: myalgic encephalomyelitis/chronic fatigue syndrome

## References

[1] Frost JH, Massagli MP (2008). Social uses of personal health information within PatientsLikeMe, an online patient community: what can happen when patients have access to one another's data. J Med Internet Res.

[2] Johnston AC, Worrell JL, Di Gangi PM, Wasko M (2013). Online health communities: an assessment of the influence of participation on patient empowerment outcomes. Info Technology & People.

[3] King SA (1996). Researching Internet communities: proposed ethical guidelines for the reporting of results. Inform Soc.

[4] Reid E (1996). Informed consent in the study of on-line communities: a reflection on the effects of computer-mediated social research. Inform Soc.

[5] Eysenbach G, Till JE (2001). Ethical issues in qualitative research on internet communities. BMJ.

[6] Elwell L, Grogan S, Coulson N (2011). Adolescents living with cancer: the role of computer-mediated support groups. J Health Psychol.

[7] Coulson NS, Buchanan H, Aubeeluck A (2007). Social support in cyberspace: a content analysis of communication within a Huntington's disease online support group. Patient Educ Couns.

[8] Rier DA (2007). Internet social support groups as moral agents: the ethical dynamics of HIV+ status disclosure. Sociol Health Illn.

[9] Seale C, Ziebland S, Charteris-Black J (2006). Gender, cancer experience and internet use: a comparative keyword analysis of interviews and online cancer support groups. Soc Sci Med.

[10] Bond CS, Ahmed OH, Hind M, Thomas B, Hewitt-Taylor J (2013). The conceptual and practical ethical dilemmas of using health discussion board posts as research data. J Med Internet Res.

[11] Markham A, Buchanan E (2012). Ethical Decision-Making and Internet Research: Recommendations from the AoIR Ethics Working Committee (Version 2.0).

[12] Daker-White G, Sanders C, Greenfield J, Ealing J, Payne K (2011). Getting a diagnosis v. learning to live with it? The case of the progressive ataxias. Chronic Illn.

[13] boyd d, Marwick AE (2011). Social privacy in networked publics: Teens' attitudes, practices, and strategies. http://www.danah.org/papers/2011/SocialPrivacyPLSC-Draft.pdf.

[14] Sveningsson M, Buchanan E (2003). Ethics in Internet ethnography. Readings in Virtual Research Ethics: Issues and Controversies.

[15] Suler J (2004). The online disinhibition effect. Cyberpsychol Behav.

[16] Bassett E, O'Riordan K Internet Research Ethics.

[17] Hogan B (2010). The presentation of self in the age of social media: distinguishing performances and exhibitions online. B Sci Technol Soc.

[18] Nissenbaum H (2004). Privacy as contextual integrity. Washingt. Law Rev.

[19] Nissenbaum H (2011). A contextual approach to privacy online. Daedalus.

[20] Marwick AE, boyd D (2014). Networked privacy: how teenagers negotiate context in social media. New Media Soc.

[21] Hudson JM, Bruckman A (2004). “Go Away”: participant objections to being studied and the ethics of chatroom research. Inform Soc.

[22] Bradley SK, Carter B (2012). Reflections on the ethics of internet newsgroup research. Int J Nurs Stud.

[23] McKee R (2013). Ethical issues in using social media for health and health care research. Health Policy.

[24] Palys T, Atchison C (2012). Qualitative research in the digital era: obstacles and opportunities. Int J Qual Meth.

[25] Department of Health (2010). Improving the Health and Well-Being of People with Long Term Conditions.

[26] Guise J, McVittie C, McKinlay A (2010). A discourse analytic study of ME/CFS (Chronic Fatigue Syndrome) sufferers' experiences of interactions with doctors. J Health Psychol.

[27] Dyer C (2006). Research in Psychology: A Practical Guide to Methods and Statistics.

[28] Jenkins N, Bloor M, Fischer J, Berney L, Neale J (2010). Putting it in context: the use of vignettes in qualitative interviewing. Qual Res.

[29] (2014). The National Archives.

[30] Mauthner N, Doucet A, Ribbens J, Edwards R (1998). Refections on a voice-centred relational method: analysing maternal and domestic voices. Feminist Dilemmas in Qualitative Research: Private Lives and Public Texts.

[31] Braun V, Clarke V (2006). Using thematic analysis in psychology. Qual Res Psychol.

[32] Basit T (2003). Manual or electronic? The role of coding in qualitative data analysis. Educ Res.

[33] Sharf BF (1997). Communicating breast cancer on-line: support and empowerment on the Internet. Women Health.

[34] Sanders C, Rogers A, Gardner C, Kennedy A (2011). Managing 'difficult emotions' and family life: exploring insights and social support within online self-management training. Chronic Illn.

[35] Papacharissi Z, Gibson P, Trepte S, Reinecke L (2011). Fifteen minutes of privacy: privacy, sociality, publicty on social network sites. Privacy Online.

[36] Bullingham L, Vasconcelos AC (2013). 'The presentation of self in the online world': Goffman and the study of online identities. J Inf Sci.

[37] Greaves F, Ramirez-Cano D, Millett C, Darzi A, Donaldson L (2013). Harnessing the cloud of patient experience: using social media to detect poor quality healthcare. BMJ Qual Saf.

[38] Shepherd A, Sanders C, Doyle M, Shaw J (2015). Using social media for support and feedback by mental health service users: thematic analysis of a twitter conversation. BMC Psychiatry.

[39] Parker AG (2014). Reflection-through-performance: personal implications of documenting health behaviors for the collective. Pers Ubiquit Comput.

[40] Goffman E (1959). The Presentation of Self in Everyday Life.

[41] Finn J (2008). Computer-based self-help groups. Soc Work Groups.

[42] Viswanath K (2011). Cyberinfrastructure: an extraordinary opportunity to bridge health and communication inequalities?. Am J Prev Med.

[43] Lee C (2009). The role of Internet engagement in the health-knowledge gap. J Broadcast Electron Media.

[44] Osatuyi B (2015). Is lurking an anxiety-masking strategy on social media sites? The effects of lurking and computer anxiety on explaining information privacy concern on social media platforms. Comput Human Behav.

[45] Nutbeam D (2000). Health literacy as a public health goal: a challenge for contemporary health education and communication strategies into the 21st century. Health Promot Int.

[46] Burkell J, Fortier A, Wong C, Simpson JL (2014). Facebook: public space, or private space?. Inform Commun Soc.

